# Scoping reviews in orthodontics: are they justified?

**DOI:** 10.1186/s40510-022-00442-3

**Published:** 2022-12-26

**Authors:** Filippos Mikelis, Despina Koletsi

**Affiliations:** 1 School of Dentistry, National and Kapodistrian University of Athens, Athens, Greece; 2 Clinic of Orthodontics and Pediatric Dentistry, Center of Dental Medicine, University of Zurich, Plattenstrasse 11, 8032 Zurich, Switzerland; 3 Meta-Research Innovation Center at Stanford (METRICS), Stanford University, Stanford, CA USA

**Keywords:** Scoping Reviews, Knowledge synthesis, Orthodontics, PRISMA ScR, Evidence synthesis

## Abstract

**Background:**

Scoping Reviews (ScRs) have emerged in the orthodontic literature as a new methodological perspective to collate and summarize scientific evidence. The aim of the present study was to identify and record the proportion of Scoping Reviews in orthodontics that have been clearly and adequately justified, based on the methodological framework of such types of reviews. Associations with a number of publication characteristics were also sought. Three major databases, namely PubMed, Scopus and Web of Science Core Collection, as well as 11 specialty orthodontic journals were electronically sought from inception until August 1, 2022, for ScRs. The primary outcome pertained to whether the published reports of the ScRs included an appropriate justification and explanation for the selection of this kind of knowledge synthesis methodology. Potential association with year, journal, continent of authorship, number of authors, methodologist involvement, appropriate reporting guidelines and registration practices followed were explored.

**Results:**

A total of 40 ScRs were eligible for inclusion, with the majority not being adequately justified (22/40; 55.0%). The majority of studies were published from 2020 onward (32/40; 80.0%). The regression model did not reveal any significant association between justification of ScRs and a number of publication characteristics (*p* > 0.05 at all levels).

**Conclusions:**

Less than half of the included ScRs were adequately justified in terms of selection of the appropriate synthesis methodology. Awareness should be raised in the scientific community regarding the correctness of the use of this newly emerging type of study in orthodontics, to safeguard against any trace of research waste.

## Background

The quality of reporting and strength of evidence perspective when examining studies and clinical trials have been instrumental in Orthodontic research [1, 2]. Reasoning for this is rather straightforward, being represented by the necessity to provide clinical decision-making toward the patients in a way that is the most beneficial and less harmful. A high degree of certainty is therefore anticipated. It is evidently well accepted that Systematic Reviews (SRs) and meta-analyses acquire the topmost position in the evidence pyramid. Conditional on their conduct, reporting and inherent bias they may provide scientists, clinicians and patients with well-documented and evidence-based knowledge in a variety of fields across biomedicine.

In hard terms, SRs are reviews that use “explicit, systematic methods to collate and synthesize findings of studies that address a clearly formulated question” [3]. They follow a structured methodology from research questions to reporting of an array of vital elements, which are currently framed under the PRISMA 2020 Statement (Preferred Reporting Items for Systematic reviews and Meta-Analyses) guidelines [4]. Because their position in the evidence base and the role and perspective SRs seem to play in commissioning the most relevant and focused answer to the questions of stakeholders, researchers, scientists, clinicians and patients, a clear understanding of how such types of studies are used compared to other types of reviews is reasonably required [5].

Lately, a number of review articles appearing as Scoping Reviews (ScR) have emerged in the dental literature, including Orthodontics, although ScR methodology has been described since 2005 in biomedicine and potentially earlier in a non-formal terminology [6]. Scoping Reviews have been described according to the typology used by Grant and Booth as performing a “Preliminary assessment of potential size and scope of available research literature,” which “aims to identify nature and extent of research evidence (usually including ongoing research)” [7]. In essence, Scoping Reviews differ on their fundamental basis from the well-known systematic reviews of the literature. The ScRs aim to gather knowledge and explore conceptual and logistic boundaries over a broad topic, with no specific focus on any particular and narrowly defined research question, with an end-role to potentially inform future SRs [8, 9].

An early empirical report regarding the utilization of ScRs to inform knowledge about evidence gaps has revealed mostly suboptimal and non-standardized methodologies followed by the authors of the ScRs in their conduct and reporting across biomedicine [10]. Interestingly, one of the most vital elements of the need to undertake a ScR is the justification of the selection of this specific type of review and its necessity to inform further research perspectives. If justification is not adequate, then the value of the ScR is likely to be under question. To this end, a very recent report in oral health has underpinned the potential lack of knowledge by the ScR authors to correctly identify the reasons that have led them to undertake such types of reviews, with nearly half failing to provide a clear rationale for choosing this route [11]. In essence, this is currently the only report on the rationale of ScRs in dentistry. The field of orthodontics has not been adequately represented in this first report, while there is an apparent rise in the number of ScRs during the last 2 years.

Therefore, it was the aim of the present empirical report to identify whether ScRs in orthodontics were adequately justified when selected, in terms of their methodological perspectives, with no timing or journal publication limitations. As a secondary aim, we examined association of ScRs justification with publication characteristics such as year of publication, journal (specialty or general dentistry), inclusion of a reporting guideline and study registration. The initiative for this work has been driven by the immense appearance of this type of study in orthodontics lately, to control any further research waste, if existent, and to support a potential improvement in their use.

## Methods

We conducted electronic searches within the following databases with no time or other restriction: MEDLINE via PubMed, Scopus and Web of Science (core collection). The date of the search was April 1, 2022; this was further updated for retrieval of new studies, on August 1, 2022. The keywords utilized included “orthodontic” and “scoping review,” following a pre-specified search strategy (“Appendix 1”). To eliminate loss of reports we further online searched all journals in the field as follows: the American Journal of Orthodontics and Dentofacial Orthopedics (AJODO), European Journal of Orthodontics (EJO), Orthodontics and Craniofacial Research (OCR), Angle Orthodontist, (ANGLE), Progress in Orthodontics, International Orthodontics, Journal of the World Federation of Orthodontists, Turkish Journal of Orthodontics, APOS Trends in Orthodontics, Seminars in Orthodontics, and Korean Journal of Orthodontics.

Studies were selected based on the inclusion of terminology indicating the conduct of a “scoping review,” either in the title, abstract or methodology section of the manuscript. Thus, full texts were screened for all studies retrieved after the search strategy was employed. Data extraction for eligible studies was conducted by two authors (FM, DK), on pre-defined standardized piloted forms, while initial calibration was conducted on 20 articles and inter-rater agreement was assessed. All included ScRs were assessed for the primary outcome by two authors. Any disagreement was settled after discussion, until a consensus was reached.

The primary outcome was defined as follows: whether the included ScRs adequately explained and justified correctly their rationale to conduct this type of review. In other words, we sought to identify whether the authors of the ScRs correctly considered and justified the conduct of the ScR as the most appropriate methodology to identify the knowledge gap and inform the research agenda in the field. Examples of adequate and appropriate justification are presented in “Appendix 2.” Additionally, a number of ScR characteristics were recorded: whether the ScR was published in a specialty orthodontic journal, year of publication, geographic region based on the affiliation details of the corresponding author, number of authors in the author list, inclusion of a methodologist (as documented by the affiliation of the authors), registration of the study and whether the ScR followed any specific reporting guidelines, according to what was reported by the authors of the ScRs.

### Statistical analysis

Descriptive statistics were performed for the pre-defined variables. Cross-tabulations were constructed to assess the association between the use of appropriate justification for the conduct of the ScR or otherwise and the aforementioned publication characteristics. Univariable and multivariable logistic regression was performed to examine the effect of year of publication, journal (specialty or general dentistry), inclusion of a reporting guideline and study registration on the primary outcome of interest. The predictors were inserted sequentially one at a time in the initial model (forward stepwise variable selection), and best-fit model selection was based on the information criteria *Akaike Information Criterion* (AIC) and *Bayesian Information Criterion* (BIC). The model which minimized the considered information criteria was selected. The unweighted kappa statistic was used to assess inter-rater agreement as per the primary outcome. A kappa value of 0.89 (95% confidence interval, CI 0.69–1.00) was achieved, denoting almost perfect agreement. The predefined level of significance was set at *p* < 0.05 (two-sided). All analyses were conducted with Stata version 15.1 (Stata Corporation, College Station, Texas, USA).

## Results

**Fig. 1:**
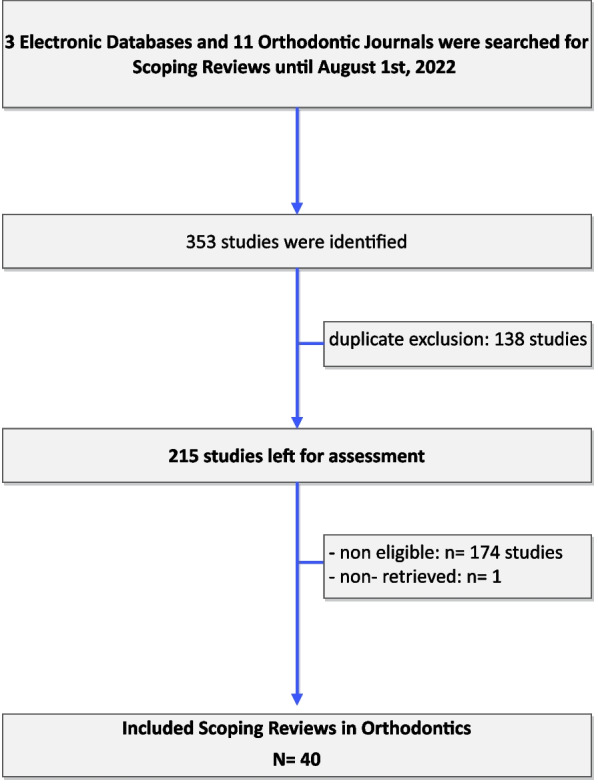
Flow diagram of study selection process

**Fig. 2:**
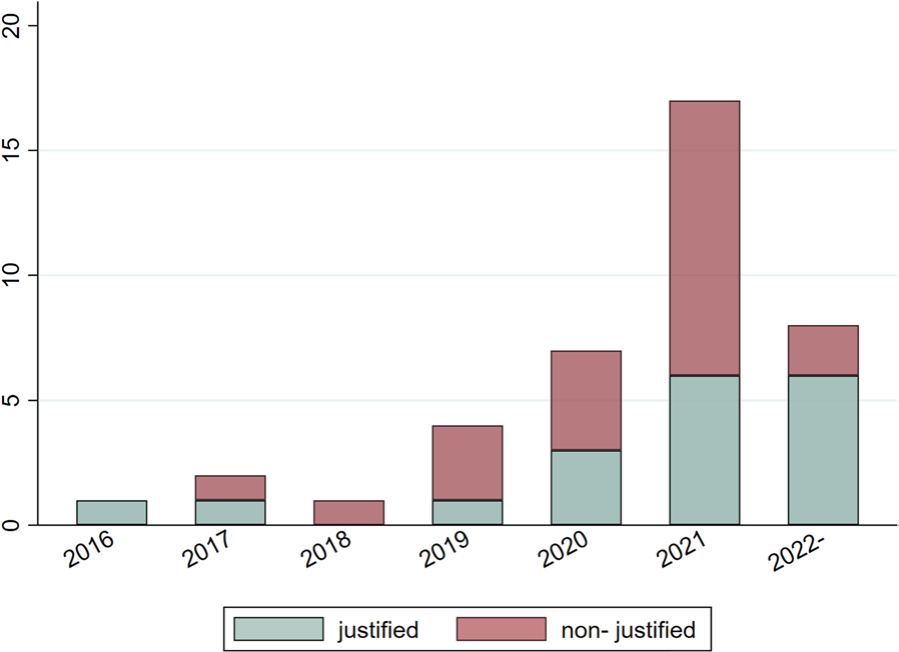
Distribution of Scoping Reviews per justification practices, across the years

The study selection process is presented in Fig. 1. A total of 40 published Scoping Reviews were eligible for inclusion. Their distribution over the years revealed a considerable rise in the number of published ScRs from 2020 onward, comprising more than 3/4 (32/40; 80.0%) of the total number of eligible studies (Table 1; Fig. 2). Most were published in orthodontic journals (22/40; 55.0%), under European (17/40; 42.5%), or other non-American (16/40; 40.0%) related to corresponding author affiliations, while the most frequent number of authors in the author list was 4–5 (18/40; 45.0%). A methodologist, according to the affiliation details of the authors, was involved only in one ScR. Only half of the included ScRs followed specific and appropriate reporting guidelines, such as the PRISMA guidelines for Scoping Reviews, or the guidance document by the Joanna Briggs Institute (21/40; 52.5%). Registration practices of a protocol of the ScR were adopted by a very limited number of studies (6/40; 15.0%) (Table 1). The most prevalent topics in the ScRs included were related to adverse effects, artificial intelligence and machine learning, as well as malocclusion prevalence and traits. The rest thematological domains were represented by one or two eligible ScRs, but were otherwise common subjects in the orthodontic literature, such as aligners, airway dimensions, white spot lesions and sleep apnea (Table 2).

**Table 1:** Characteristics of Scoping Reviews (ScRs), by adequate justification for their conduct (*n* = 40)

	Justification for conducting Scoping Reviews		
	No *N* (%)	Yes *N* (%)	Total *N* (100%)
Year			
2016	0 (0.0)	1 (100.0)	1
2017	1 (50.0)	1 (50.0)	2
2018	1 (100.0)	0 (0.0)	1
2019	3 (75.0)	1 (25.0)	4
2020	4 (57.1)	3 (42.9)	7
2021	11 (64.7)	6 (35.3)	17
2022	2 (25.0)	6 (75.0)	8
Journal			
Orthodontic	11 (50.0)	11 (50.0)	22
Non-specialty	11 (61.1)	7 (38.9)	18
Continent			
America	1 (14.3)	6 (85.7)	7
Europe	10 (58.8)	7 (41.2)	17
Asia/other	11 (68.7)	5 (31.3)	16
No. of authors			
1–3	8 (61.5)	5 (38.5)	13
4–5	9 (50.0)	9 (50.0)	18
≥ 6	5 (55.6)	4 (44.4)	9
Methodologist			
No	22 (56.4)	17 (43.6)	39
Yes	0 (0.0)	1 (100.0)	1
Appropriate reporting guidelines [if any]			
No	13 (68.4)	6 (31.6)	19
Yes	9 (42.9)	12 (57.1)	21
Registration			
No	18 (52.9)	16 (47.1)	34
Yes	4 (66.7)	2 (33.3)	6
Total	22 (55.0)	18 (45.0)	40

**Table 2:** Distribution of topics related to the included Scoping Reviews

Topics	*N*	%
Adverse effects [trauma, root resorption]	6	15.0
Artificial intelligence and machine learning	5	12.5
Malocclusion prevalence and traits	3	7.5
Bruxism and temporomandibular disorders	2	5.0
Cleft lip and palate	2	5.0
Core outcome set	2	5.0
Orthodontics and COVID-19	2	5.0
Patient-reported outcomes	2	5.0
Periodontal ligament properties and mechanics	2	5.0
Recent advances in orthodontics	2	5.0
White spot lesions	2	5.0
Aesthetic perception	1	2.5
Airway dimensions	1	2.5
Aligners	1	2.5
General medicine and orthodontics	1	2.5
Mini-implants	1	2.5
Orthodontic materials	1	2.5
Orthognathic surgery	1	2.5
Sleep apnea	1	2.5
Smartphone applications in orthodontics	1	2.5
Surgical tooth acceleration interventions	1	2.5
Total	40	100

Overall, 18 ScRs (45.0%) reported clear and adequate justification for the following scoping review methodology, with the majority being non-appropriately justified and explained (22/40; 55.0%). According to the regression model (Table 3), there was no evidence that year, journal, utilization of appropriate guidelines or registration practices were significantly associated with adequate justification for the conduct of ScRs (*p* > 0.05 at all levels).

**Table 3:** Univariable and multivariable logistic regression with odds ratios (ORs) and respective confidence intervals (CIs) for the effect of year, journal, appropriateness of reporting guidelines used and registration, on adequate justification for the conduct of a scoping review

Category	Univariable			Multivariable*		
	OR	95% CI	*p *value	OR	95% CI	*p* value
Year						
Per unit	1.12	0.72, 1.75				
Journal						
Non-specialty	Reference					
Orthodontic	0.64	0.18, 2.25				
Appropriate reporting guidelines			0.11			0.11
No	Reference			Reference		
Yes	2.89	0.79, 10.58		2.89	0.79, 10.58	
Registration						
No	Reference					
Yes	0.56	0.09, 3.49				

*The presented model (in essence univariable) indicated the lowest values of AIC and BIC information criteria

## Discussion

The findings of the present report highlight the potential lack of knowledge and experience by researchers and authors in the orthodontic field, aiming to conduct a review with a scoping methodology perspective. It is concerning that about half of the currently existing ScRs about orthodontics, irrespective of the journal of publication and other predictors, fail to provide a clear justification for the selection of this methodological route to map existing evidence; the concurrent impact on the identification of knowledge gaps and/or contribution to the recognition of areas and domains in need of further study is profound.

In essence, suboptimal knowledge of research methodology and reporting issues is not new to both dental and orthodontic literature. Previous studies on the reporting quality of systematic reviews in orthodontics have indicated several domains of non-standard, transparent and consistent methodological perspectives being followed [12–15]. Moreover, it has been reported that a quarter of primary research studies do not correctly identify a relevant systematic review of a topic to be adequately justified [16].

Evidence on Scoping Reviews, especially regarding dentistry and orthodontics, is rather recent. The sole empirical study on the justification of conducting ScRs in dentistry was that of Zauza et al. [11], which informed the literature in the field about the necessity for improvement in justification practices, by the authors of the ScRs, when deciding to undertake such an initiative. This report included ScRs from various dentistry specialty domains, and overall, the investigators concluded that only half of the examined studies correctly identified and justified why they followed this type of review methodology [11]. It was notable that ScRs related to orthodontics in this study constituted only a small fraction of the sample, contributing to approximately 4% of it, or 7 studies in absolute number. Furthermore, the vast majority of ScRs in oral health represented in this first report were held in the disciplines of Dental Public Health, Paediatric Dentistry, Oral and Maxillofacial Surgery and Pathology and Periodontology, thus leaving orthodontics lagging far behind; although no differences between the groups of dental scientific fields were identified, this cannot preclude effects of inadequate power assumptions stemming from underrepresentation of specific domains. Only a fourth to a tenth of the identified ScRs by Zauza et al. were found to be associated with an existing protocol and preregistration practices. The latter was apparently in agreement with the findings of the present study, where we identified that only 16% of the ScRs in orthodontics were previously registered. Registration practices have been reported to bear implications regarding research integrity, clarity and transparency overall and have been associated also with other research design and conduct flaws across the dental and orthodontics literature [17–19].

Lately, an extension to PRISMA guidelines for Scoping Reviews has been developed in order to facilitate the completeness and transparency in reporting the increasing number of this type of reviews appearing in the biomedical literature [20]. This document has followed prior but not systematic efforts to map scoping review methodology [21, 22], as determined by the Joanna Briggs Institute guidance document. It highlights the importance of increasing the level of complete and accurate reporting of this type of knowledge synthesis, as well as their relevance to decision-making. Of the key elements of this approach is the clear identification and description of rationale by the authors, in the context of the justification and relevance of the selection of this approach. It has been reported that researchers and authors aiming to conduct a ScR often fail to distinguish the differences from systematic reviews of the literature, or potentially misinterpret scoping methodology framework as the one used for the well-known and established systematic reviews, thus, resulting in a final document of an uncertain or questionable value [23]. It has also been argued that authors select to “promote” their research as a scoping framework document, in order to skip the internal validity assessment of the included studies in the form of a risk of bias assessment, for reasons of ignorance, apparent simplicity in conducting and reporting their findings, to expedite the whole process or for other reasons [24]. This might pose additional concerns if one considers the apparent cross-linking of evidence synthesis to clinical recommendations for decision-making; for example, no clinical recommendations may be considered reliable in order to provide a recommendation of an intervention for clinical practice, when based on synthesized evidence from a ScR without any further assessment of the quality and risk of bias of the included and contributing studies.

Our results are indicative of the status of the very recent and most probably almost all published ScRs in orthodontics. The findings showcase the need to improve and advance education, knowledge and expertise of the scientific community in this field, including authors, journal editors, reviewers and stakeholders, so as to avoid a considerable portion of research waste [25]. The strength of the present meta-epidemiological study lies on the fact that it constitutes the first empirical approach to search preliminary evidence on the conducted, reported and published ScRs in orthodontics; although no further associations with potential predictor factors could be established, possibly due to power assumptions and limitations, it follows that publication of the existing ScRs in the field was at least non-adequately justified. We followed an all-inclusive approach and search strategy covering three international databases supplemented with electronic searching within eleven journals in the specialty, with no other filters or restrictions. The sample that constituted our pool of examined ScRs is considered adequate, although not large, and probably frames all evidence that exists in the field, with the vast majority of the included studies being published over the last 2 years. For this reason, it follows that the rationale for the present study is evidently well justified in order to expose the state-of-the-art of the ScRs in orthodontics and provide a twofold basis for investigation: first, facilitate early assessment of evidence on this type of knowledge synthesis and also pledge for action in the direction of early improvement in the field prior to massive publication rates; second, further in-depth examination of the reporting quality of the existing ScRs in the field, according to the PRISMA ScR guidelines.

## Conclusions

Our findings suggest suboptimal justification of Scoping Reviews appearing in the orthodontic literature. This raises concerns regarding lack of expertise in the field, while it seems critical that awareness is increased on enhancing the education and training of investigators regarding this method of knowledge and evidence synthesis.

## Data Availability

The datasets used and analyzed in the present study are available from the corresponding author upon reasonable request.

## Appendix 1: Search strategy for study selection, for MEDLINE (via PubMed) and adapted for the other databases.

Date: April 1, 2022, and updated August 1, 2022

All fields

No filters, or language/time restriction

1. Orthodontic
2. Tooth movement
3. Root resorption
4. Maxillary expansion
5. Fixed orthodontic appliances
6. Orthodontic aligners
7. Tooth agenesis
8. Class I malocclusion
9. Class II malocclusion
10. Class III malocclusion
11. Mandibular advancement
12. Maxillary protraction
13. 1 OR 2 OR 3 OR 4 OR 5 OR 6 OR 7 OR 8 OR 9 OR 10 OR 11 OR 12
14. Scoping review
15. Mapping review
16. 14 OR 15
17. 13 AND 16

## Appendix 2: Example statements of appropriate and non-appropriate justification of conducting a scoping review from the included orthodontic articles.

**Table:**

Non-appropriate justification	Appropriate justification
“The primary goal of the present article was to identify and summarize the biomarkers assessed through noninvasive methods and their correlation with radiographic maturity indicators” [26]	“Scoping Reviews are of particular use when a body of literature has not yet been comprehensively reviewed or exhibits a large, complex, or heterogeneous nature not amenable to a more thorough systematic review […] No study has attempted to systematically organize the existing literature to review existing AI and ML applications in orthodontics […] Hence, this scoping review aims to provide an overview of the existing evidence of how far the earlier AI and ML advancements in orthodontics have translated into clinical fruition.” [29]
“This scoping review is aimed at mapping the existing technological robotic applications in orthodontics as reported in orthodontic literature in the last decade” [27]	“A scoping review of literature was chosen as the method of investigation because the study question was broad and the answer involved evidence on brand new techniques which are not yet common in clinical practice.” [30]
“This scoping review was conducted to document and report in depth, the various causes, diagnosis and management of IAN damage secondary to orthodontic treatment.” [28]	“Scoping Reviews become especially beneficial when conducted on novel topics with fast-evolving evidence, in which a scarcity of randomized controlled trials (RCTs) prevent systematic reviews from having meaningful conclusions. This is such a case for mobile apps in orthodontics. This scoping review therefore aims to determine the scope and extent of the published literature on mobile apps in orthodontics, identify the types of studies published, and summarize the outcomes studied.” [31]

## Abbreviations

AIC: Akaike information criterion
AJODO: American Journal of Orthodontics and Dentofacial Orthopedics
ANGLE: Angle Orthodontist
BIC: Bayesian information criterion
CI: Confidence interval
EJO: European Journal of Orthodontics
OCR: Orthodontics and Craniofacial Research
PRISMA: Preferred Reporting Items for Systematic reviews and Meta-Analyses
ScR: Scoping Review
SR: Systematic Review
